# In-silico core proteome analysis for chimeric vaccine development against tick-borne tularemia

**DOI:** 10.1371/journal.pone.0337692

**Published:** 2025-12-26

**Authors:** Bader S. Alotaibi, Fatiha Khan, Muhammad Bilal Iqbal Rehmani, Fizza Arshad, Muhammad Umer Khan, Umar Nishan, Abid Ali, Khaled Fahmi Fawy, Sarah A. Altwaim, Saeed M. N. Alasmari, Hanna Dib, Mohibullah Shah

**Affiliations:** 1 Department of Clinical Laboratory Sciences, College of Applied Medical Sciences, Al- Quwayiyah, Shaqra University, Riyadh, Saudi Arabia; 2 Department of Biochemistry, Bahauddin Zakariya University, Multan, Pakistan; 3 Institute of Molecular Biology and Biotechnology, The University of Lahore, Lahore, Pakistan; 4 Department of Chemistry, Kohat University of Science and Technology, Kohat, Pakistan; 5 Department of Zoology, Abdul Wali Khan University Mardan, Mardan, Pakistan; 6 Department of Chemistry, Faculty of Science, Research Center for Advanced Materials Science (RCAMS), King Khalid University, Abha, Saudi Arabia; 7 Department of Clinical Microbiology and Immunology, Faculty of Medicine, King Abdulaziz University, Jeddah, Saudi Arabia; 8 Special Infectious Agents Unit, King Fahd Medical Research Center, King Abdulaziz University, Jeddah, Saudi Arabia; 9 Department of Biology, Faculty of Science and Arts, Najran University, Najran, Saudi Arabia; 10 College of Engineering and Technology, American University of the Middle East, Kuwait; 11 Department of Animal Science, Federal University of Ceara, Fortaleza, Brazil; Cholistan University of Veterinary and Animal Sciences, PAKISTAN

## Abstract

Tularemia is an extremely contagious zoonotic illness resulting from infection with the intracellular bacterium *Francisella tularensis*. It is transmitted primarily via vector bites particularly from ticks, flies, and mosquitoes and is a severe public health threat. Because of its high virulence, low infective dose, aerosol transmissibility, and potential for mass casualties, *F. tularensis* is also considered a potential biological warfare agent. Despite its severity, there is presently no licensed vaccine against this pathogen. In the present work, a subtractive proteomics pipeline was implemented to identify potential antigenic targets to prepare a multi-epitope vaccine. Five vaccine constructs were generated through the combination of B-cell, HTL, and CTL epitopes with suitable adjuvants and linkers. Among these, two constructs V1 and V2 were extremely non-allergenic and antigenic. To assess immune receptor engagement, molecular docking was conducted with TLR4 and TLR5, followed by 200 ns molecular dynamics simulations. Vaccine-receptor complexes were analyzed using RMSD, RMSF, radius of gyration (Rg), Dynamic Cross-Correlation Matrix (DCCM), SASA, PCA, H-bond analysis and MMPBSA binding energy calculations, all confirming structural stability and strong binding affinity. *In-silico* cloning revealed a GC content of 50% and 1.0 codon adaptation index (CAI), suggesting high expression potential in *E. coli*. Immune simulation further supported the construct`s ability to elicit a robust and long-lasting immunity. These computational findings highlight the potential of the constructed vaccines as effective candidates against *F. tularensis*, though experimental substantiation is requisite.

## Introduction

Tularemia is a zoonotic illness brought about by the encapsulated, nonmotile, gram-negative coccobacilli bacterium *Francisella tularensis* [1]. The *F. tularensis* species include four subspecies, each of which is differentiated by its metabolic properties and pathogenicity: *F. tularensis* subsp. *mediasiatica, F. tularensis* subsp. *tularensis*, *F. tularensis* subsp. *Novicida,* and F. *tularensis* subsp. *Holarctica* [2]. Human tularaemia is caused by two of these subspecies, *tularensis* (type A strains) and *holarctica* (type B strains) [3]. *F. tularensis* may spread from infected animals to humans through several ways. The severity of the sickness depends on the portal of entrance, infectious dosage, and biovar of the infecting strain. Tularemia in humans appears in six signs and symptoms based on the location of bacterial invasion: pulmonary, oculoglandular, ulceroglandular, glandular, oropharyngeal, and typhoid [4]. Glandular and ulceroglandular forms are the most common and are transmitted by the bites of arthropods (e.g., mosquitoes, ticks, and flies) and are responsible for 60–70% of cases of tularaemia [5].

*F. tularensis* subspecies tularensis has been considered a bioweapon candidate due to its high virulence, low infectious dosage, facile aerosol transmission, and potential to induce serious illness and mortality [6]. *F. tularensis* has been known to be extremely infectious since the mid-20th century, as evidenced by waterborne epidemics in the Soviet Union and Europe, laboratory mishaps, and epizootic cases in the US [7]. In 1966–1967, Sweden witnessed the greatest naturally arising outbreak of aerosol tularemia, infecting almost 600 farmers with F. tularensis type B [8]. Research groups from Japan conducted research on *F. tularensis* subsp. *tularensis*, which potentially was used against Russian soldiers, Chinese civilians, and American prisoners of war from 1932 to 1945 [9]. In 1969, a World Health Organization specialist commission projected that aerial dispersion of 50 kg of infectious *F. tularensis* bacteria among a five-million-person metropolitan region would cause 250,000 cases requiring severe medical care and 19,000 deaths [6].

Commonly prescribed antibiotics for tularemia include fluoroquinolones (e.g., ciprofloxacin), aminoglycosides such as gentamicin and streptomycin, and tetracyclines (e.g., doxycycline) [10], but *F. tularensis* is becoming resistant to these drugs [11]. Vaccines activate the immune system and protect against certain infections. However, currently, no effective and safe vaccine is available to combat infectious *F. tularensis*. That is why there is an immediate requirement to create an efficient and successful potential vaccine against *F. tularensis.* Developing a vaccine for commercial distribution requires extensive clinical trials and significant financial investment, making it a lengthy process. Immunoinformatics can significantly shorten vaccine development time compared to traditional methods. In silico techniques have been successful in developing vaccine candidates. Recent advances in immunoinformatics, as well as the availability of a wide range of tools for designing epitope vaccines, have greatly accelerated research into the development of innovative potential vaccine candidates [12]. For this purpose, this study prioritized subunit vaccine candidates from *F. tularensis’s* core proteome using subtractive proteomics and then applied immunoinformatics analysis to forecast the B and T-cell epitopes. Two vaccine constructs were prioritized from the five designed vaccine constructs based on various physicochemical properties. The MEV constructs’ affinity for the TLR4 and TLR5 receptors was ascertained using docking and simulation experiments. Additionally, conformational changes in the receptor and MEV that impact construct binding were sought. A vector model of *Escherichia coli* may effectively express the selected vaccine designs. The vaccine designs have been evaluated for interactions with human immunological receptors and completely validated using comprehensive molecular dynamics (MD) simulations. The vaccine developed in this research study holds the potential for further in vitro and in vivo testing to aid in the formulation of an effective treatment against resistant *F. tularensis*.

## Methodology

**Fig 1:**
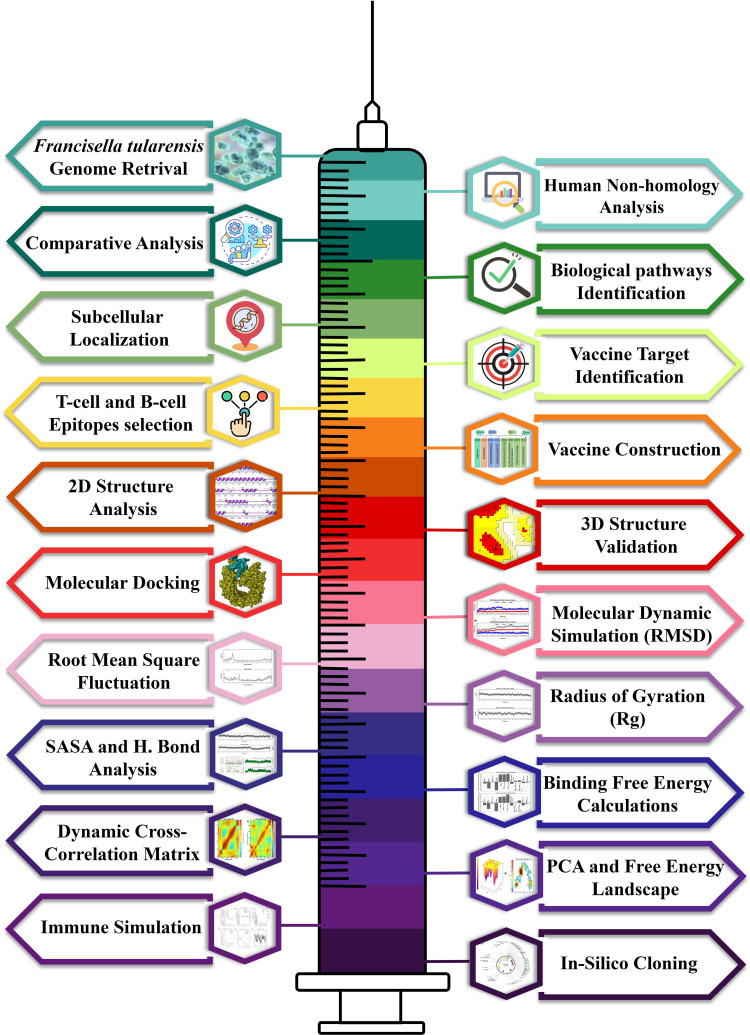
Schematic illustrations of the methods used to develop the multi-epitope-based vaccine candidate to fight against *F. tularensis.*

The following figure (Fig 1) shows the schematic diagram of all the techniques used in this investigation.

### Retrieval of core proteome

Seventy complete genomes of *F. tularensis* were downloaded from the NCBI dataset in FASTA format. The core genome was prepared using the EDGAR server using *F. tularensis* subsp. novicida (GenBank ID: GCA_000833355.1) as a reference genome. To choose possible vaccine candidates, the proteome was subsequently exposed to subtractive proteomics and reverse vaccinology techniques [13].

### Screening for Human non-homologs

After predicting the core genome, the investigation expanded to sort out the human non-homologs. The study was carried out using a standalone BLASTp against the entire human proteome database, which was obtained from the Uniprot web service. The threshold parameters of bitscore < 100, percentage identity < 35%, e-value > 1e-4, and query coverage < 35% were applied [14]. The resulting non-homologous proteins against humans were passed to a gut microbiome database via a Blastp analysis: bitscore < 100, percentage identity < 35%, e-value > 1e-4, and query coverage < 35% to identify the gut non-homologous proteins, since it will trigger an autoimmune response [15].

### Identification of virulent and essential proteins

Virulent factor proteins play a vital role in pathogens that infect the host organism; hence, the detection of such factors is equally essential for finding therapeutic targets [16]. The proteins of the pathogen were scanned against the Virulence Factor Database (VFDB) using standalone BLASTp with the threshold values: e-value < 1e-4 and bitscore ≥ 100 [17]. Moreover, essential genes play a crucial role in survival pathways, making them potential therapeutic targets. The study determines the group of essential proteins necessary for pathogen survival.

Essential proteins were filtered through the BLASTp analysis against Database of Essential Genes (DEG) with cutoff values (i.e., identity ≥35%, e-value < 1e-4, and bitscore ≥100) [18]. Selected protein targets were merged from all these screenings.

### Determination of metabolic pathways

*F. tularensis* pathways were identified using the Kyoto Encyclopedia of Genes and Genomes (KEGG) [19]. Through the KEGG server, the proteins associated with these metabolic pathways were also determined. KAAS offered the functional annotation of these proteins via the metabolic pathways. Pathogens and the host’s pathways were compared to assess the common and distinct metabolic pathways in the pathogen. KEGG orthology (KO) identifiers were returned by the KAAS server for the assignment of the KO numbers to proteins. Common and distinct pathway proteins were classified, and those proteins involved in distinct pathways of pathogens were taken forward for further analysis. The proteins with designated KO numbers were considered KEGG-dependent proteins, and other proteins were considered KEGG-independent proteins.

### Identification of the subcellular localization

Proteins may be located in multiple positions in a cell, such as the inner membrane, periplasmic membrane, outer membrane, and cytoplasm, as per subcellular localization predictions. Proteins were classified as vaccine or drug targets depending on their localization, since the membrane proteins were chosen to be the vaccine target proteins, while the cytoplasmic proteins were chosen to be the drug target proteins.[17]. For potential vaccine or drug target identification, PSORTb v3.0 and CELLO v2.5 servers predicted the subcellular localization of the proteins.

### Screening of antigenic vaccine target proteins

The membranous proteins were further analyzed for various analyses such as antigenicity, topology, and allergenicity. VaxiJen server v2.0 [20] was used to analyze and evaluate antigenic proteins, and allergenicity was determined by the AllerTOP v.2.0 server [21]. TMHMM server predicted the topology of transmembrane proteins as 0 or 1. Different physicochemical properties, including the molecular weight, aliphatic index, query length, instability index, GRAVY (Grand Average of Hydropathicity) value, and theoretical pI, were assessed by the ExpasyProtParam tool [22].

### T-cell and B-cell epitope retrieval and analysis

The Immune Epitope Database (IEDB) server predicted the T-cell epitopes (Cytotoxic T lymphocytes and Helper T lymphocytes) of the probable vaccine candidate [23]. The resulting epitopes were ranked on the basis of their percentile ranks <0.4. Cytotoxic T lymphocytes and Helper T lymphocytes epitopes were retrieved by using 2.22 method recommended by the IEDB [24]. To build a potential vaccine, particular antigens were recognized by cytotoxic T-cell receptors (TCRs). Helper T cells play a condemnatory function in the immune response, directing CTL cells to kill off the parasitized target cells and macrophages to engulf the pathogen [25]. The Bepipred linear epitope evaluation tool from the IEDB repositories predicted B-cell epitopes. B-cell epitopes identified by B-cell receptors against antigen-associated immunoglobulin were generated; hence, B-cell epitopes serve a key role in immune system adaptation [26]. Finally, toxicity, antigenicity, and allergenicity were estimated through ToxinPred, VaxiJen v2.0, and AllerTOP v.2.0 servers, respectively.

### Multi-epitope vaccine formation

The novel multi-epitope vaccines were formulated via fusion of adjuvant, B-cell, and T-cell epitopes with the respective linkers. An adjuvant is an immune-stimulating reagent that boosts the effectiveness of a vaccine and requires precise selection, since multi-epitope vaccines alone tend to have low immunogenicity [27]. Through α-helical EAAAK linker five adjuvants were added to the vaccine sequence, including flagellin, beta-defensin, granulocyte-macrophage colony, HBHA-conserved adjuvant, and HBHA adjuvant. The EAAAK linker provides sufficient domain separation within a bifunctional fusion protein and is utilized to bridge the adjuvant to the first CTL epitope. Linkers are required to maintain the functional integrity of composite epitopes [28]. GPGPG and AAY linkers were utilized to couple HTL (Helper T lymphocytes) and CTL (Cytotoxic T lymphocytes) epitopes, respectively. To maintain their inherent immune-stimulating character, LBL (Linear B Lymphocytes) epitopes were coupled with bi-lysine (KK) linkers [29].

**Assessment of Designed Vaccines**The MEVs were investigated for physicochemical properties through the ProtParam server. Physicochemical characteristics included theoretical isoelectric point, molecular weight, aliphatic index, and GRAVY (Grand Average of Hydropathicity) value [30]. Further, the VaxiJen v2.0 server calculated the antigenicity score of the vaccines [20]. Furthermore, the server AllerTOP v.2.0 [21] examines the vaccines’ allergenicity with the primary objective of preventing any allergen responses caused by vaccination [26].The constructs’ solubility was checked using a sequence-based prediction approach by the SOLpro server [31].

### 2D and 3D structure analysis of vaccine constructs

SOPMA and PDBsum predicted the 2D structure of the developed vaccines [32]. This investigation also evaluated other features, including α-helices, extended chains, random coils, and the degree of beta turns [33]. Furthermore, trRosetta predicted three-dimensional structures of the constructs [34]. The tertiary structure was validated using ERRAT (quality 90%) and ProSA-web. ProSA-web is frequently applied to find prospective defects in 3D structures. The PROCHECK service was utilized to acquire the Ramachandran plot [35]. The phi/psi angles were analyzed via Ramachandran plot and provide a thorough understanding of protein structure.

### Identification of globular and disordered regions of vaccine constructs

Functional proteins rely on the well-characterized globular structure. Most globular proteins consist of and act as modules that contain disordered pieces like domain ligands and post-translational modification spots [36]. Globplot2 server was employed for the determination of the globular and disordered parts of the constructs.

### Post-translational Analyses of the prioritized vaccine constructs

During the development of vaccines, post-translational modifications regulate the functional activity of proteins, inducing immunogenicity and stability [37]. In order to check whether our vaccine constructs could undergo post-translational modifications, NetPhos 3.1 [38] evaluated the phosphorylation locations of the selected vaccine constructs, and the NetNGlyc 1.0 server [39] identified N-glycosylation sites in the constructs.

### Detection of Conformational B-cell Epitopes

Protein folding creates discontinuous or conformational B-cell epitopes through the combination of residues located far from each other to form a distinct 3D form. The 3D structure of the discontinuous B-cell epitopes in the chosen multi-epitope vaccine constructs was assessed using the ElliPro server [40] and DiscoTope 3.0 [41]. Moreover, DiscoTope-3.0 is a significantly improved conformational B-cell epitope prediction tool that use an inverse folding structure representations and a positive-unlabelled learning technique, and is adaptable for both solved and predicted structures [41].

### Prediction of binding pocket

The binding pocket or ligand-binding site analysis provides a good understanding of molecular docking and its processes. Protein structures are very complex with a number of internal cavities, surface pockets, and cross-channels. The structural basis and microenvironments presented by these topographic features make it possible for proteins to perform various functions like enzymatic activity, ligand binding, and DNA association. Therefore, identification and study of the topographical features of proteins are necessary to ascertain the relationship between protein structure and function, as well as to design therapeutics that can target these proteins effectively [37]. The Computer Atlas of Surface Topography of Proteins (CASTp) service identified the binding pockets in the vaccine constructs [42]. The default probe radius of 1.4 Ǻ was applied.

### Molecular docking

A productive immunological reaction is created when the vaccine interacts with the host immune system cells. Molecular docking analysis determined the ability of the prioritized MEV constructs (V1 and V2) to bind to human immunological receptors TLR4 and TLR5 respcetively. The ClusPro 2.0 server was utilized to dock MEV constructs (V1 and V2) with TLR4 and TLR5. ClusPro 2.0 server predicts interactions between two protein structures, visualized by pymol [43].

### Molecular dynamics simulations

The binding pattern and interaction assessment between the vaccine and human toll-like receptors (TLR4, TLR5) were investigated using the molecular dynamics simulation for a time period of 200 ns. For this purpose, to check the vaccine’s stability and flexibility, energy minimization and molecular dynamics (MD) simulations were assessed through the AMBER 20 software program, as well as to offer light on how the vaccine model interacts inside a biological system [44]. To study the dynamic parameters, the protein was placed within the LEaP system and subjected to ff14SB amber settings. At the same time, the ligand was subjected to a generalized AMBER force field. The LEaP system protonated proton-containing proteins using the counterion 2Cl-. The complex from LEaP with a margin distance of 9.0 Å was solved using SPCBOX. Upon system stabilization, Molecular dynamics (MD) simulations were run for 200 ns through the NPT ensemble at 1 atm pressure and 300 K temperature [45].

### MD trajectory analysis

The AMBER 20 program were utilized to analyze the MD trajectory. The system setup, analysis involved the 03 steps of the simulation. In the step of trajectory analysis, several trajectories were produced: solvent accessible surface area (SASA), root mean square deviation (RMSD), radius of gyration (Rg), hydrogen bond analysis (H-bond analysis), Dynamic Cross-Correlation (DCCM) analysis and root mean square fluctuation (RMSF). The protein structure’s time-dependent fluctuation is measured by the RMSD, while RMSF measures residue-wise variations. Additionally, the compactness and relaxation of the complexes were investigated using the Rg, since structural compactness is essential for preserving the stability of the complex [46]. SASA analysis was conducted to estimate the amount of protein surface available to the solvent by quantifying the distribution of hydrophilic and hydrophobic residues [47]. The H bonds between the vaccine and the TLR were examined using the relevant index files for each chain. Dynamic Cross-Correlation (DCCM) analysis determines the correlated and anti-correlated motions between vaccine and TLR receptor. 

### Binding free energy calculation

By using snapshots captured through MD simulation, energetic affinities were computed in order to examine the conformational and thermodynamic characteristics of the complexes. A molecular mechanics-based method, including the MMPBSA module incorporated into AMBER20, was utilized to determine binding free energy [45]. It can be computed as follows:


$$\Delta {𝐆}_{𝐛𝐢𝐧𝐝}=\;\Delta {𝐆}_{𝐫𝐞𝐜𝐞𝐩𝐭𝐨𝐫+\;𝐋𝐢𝐠𝐧𝐚𝐝}\;-\;(\Delta {𝐆}_{𝐑𝐞𝐜𝐞𝐭𝐨𝐫}+\Delta {𝐆}_{𝐋𝐢𝐠𝐚𝐧𝐝})$$ (1)

Molecular mechanics energy fragmentation was probed as non-bonded electrostatic energies (Eele), vdW energies (EvdW), and the free solvation energy (Gsol), which consisted of both polar and nonpolar solvation energies.


$$\Delta {𝐆}_{𝐬𝐨𝐥}\;=\;\Delta {𝐆}_{𝐞𝐥𝐞,𝐬𝐨𝐥𝐏𝐁(𝐆𝐁)}\;+\;\Delta {𝐆}_{𝐧𝐨𝐧𝐩𝐨𝐥,𝐬𝐨𝐥}$$ (2)

The AMBER20 MMPBSA free energy decomposition algorithm was used to get valuable insight into the contributions of important residues involved in ligand-protein binding. The ligand-receptor complex interaction term can be further classified into the polar (ΔGele,sol), non-polar (ΔGnonpol,sol), electrostatic (ΔGele), and vdW (ΔGvdW) contributions [48].


$$\Delta {𝐆}_{𝐯𝐚𝐜𝐜𝐢𝐧𝐞–\;𝐫𝐞𝐜𝐞𝐩𝐭𝐨𝐫}\;=\;\Delta {𝐆}_{𝐯𝐝𝐖}+\;\Delta {𝐆}_{𝐞𝐥𝐞\;}+\;\Delta {𝐆}_{𝐞𝐥𝐞,𝐬𝐨𝐥}+\;\Delta {𝐆}_{𝐧𝐨𝐧𝐩𝐨𝐥,𝐬𝐨𝐥}$$ (3)

### PCA analysis and Free energy landscape

By computing eigenvectors and eigenvalues and projecting the first two principal components, Principal Component Analysis (PCA), a familiar unsupervised dimensionality reduction technique, describes changes in the collective energy landscape of MD trajectory data. It also simplifies the data complexity and selects the different modes engaged in the protein movement. To create the covariance matrix, the principal components (PC1, PC2) corresponding to the covariance matrix feature vectors were computed. The free energy landscape study used PC1 and PC2, which are the coordinate axes obtained from principal component analysis (PCA) of the docking complex’s RMSD and Rg values. A three-dimensional map of the free energy landscape was created using relative free energy as the Z-axis [49].

### In-silico cloning and immune simulation of the constructs

The Java codon adaptation tool [50] was used to transform codons in vaccine constructs based on the expression system. The multi-epitope vaccine constructs were reverse-transcribed into a DNA sequences in the presence of Codon Adaptation Index (CAI) optimization. Unwanted components like restriction enzyme recognition sites, ribosome binding sites, and rho-independent transcription terminators were removed to promote efficient expression. The SnapGene software was utilized to model the cloning of the constructs into the pET28a(+) vector for expression in an *E. coli* host. The C-ImmSim service was used to simulate immunological responses and evaluate vaccine efficacy and immunological profile [51]. The immunological simulation settings included volume (10), number of steps (1000), random seed (12345), and total injections (01, 84, and 168 hr). As per established practice, recommended gap between two doses is four weeks and were applied in triplicate here.Simulation duration was calculatedove time with each step as 8 hours. So, 01, 84, and 168 hr translate to 672 hours (≈4 weeks). The remaining parameters were left as defaults.

### Immunological coverage assessment

HLA allele prevalence and geographic spread differ across ethnic backgrounds and regions that affect vaccine result [52]. The IEDB population coverage tool assessed the population coverage, where defined MHC1 and MHC2 epitopes and HLA-binding alleles were counted [53]. Based on the distribution of human MHC binding alleles, this server predicts the population coverage of each epitope for various places throughout the world [29].

## Results

### Core proteome retrieval

Seventy-two *F. tularensis* proteomes were downloaded from the NCBI database and evaluated using the EDGAR database to construct the core proteome. The EDGAR database yielded 619 core proteins, later utilized for further subtractive proteomics and reverse vaccinology analysis.

### Human non-homolog protein analysis

To find new targets, bacterial proteins were filtered based on similarity with host proteins. After discarding homologous proteins, 540 human non-homologous proteins were identified by screening against the human proteome database. The gut microbiota colonizes the human host and carries out a number of metabolic reactions, such as defense, homeostasis, physiological functions and growth. To reduce the risk of an autoimmune response, key proteins were scanned against the gut microbiome using Blastp analysis, which resulted in 160 gut non-homologous proteins.

### Essential and virulent protein identification

Essential genes are indispensable to survival and perform basic biological functions. DEG database was utilized to determine these proteins of the pathogen. 64 proteins were identified as essential for maintaining cellular functioning in *F. tularensis*. The virulence proteins were analyzed by VFDB. VFDB identified 15 proteins as virulence factors through BLASTp analysis. A total of 79 essential and virulent proteins were identified, and after the removal of duplicates, 77 proteins were processed for downstream analysis.

### Pathogen metabolic pathway analysis

The KEGG queried 345 metabolic pathways for humans and 95 for *F. tularensis*. Manual pathway comparison of the human and bacterial metabolic pathways identified 23 pathways found only in bacteria that were not present in human pathways (Table 1), whereas 72 pathways were common in both. Moreover, proteins involved in unique pathways were also identified. The proteins were allocated a KO (KEGG ortholog) by the KAAS server. From 77 proteins found in earlier steps, 41 proteins got their KO numbers and were identified as KEGG-dependent proteins and the rest of 36 proteins were categorized as KEGG-independent, but only 16 out of 41 were involved in pathogen-specific pathways.

**Table 1:** List of *F. tularensis* distinctive patways.

Sr. No.	Accession	Pathway name
1.	ftu00261	Monobactam biosynthesis
2.	ftu00300	Lysine biosynthesis
3.	ftu00362	Benzoate degradation
4.	ftu00460	Cyanoamino acid metabolism
5.	ftu00521	Streptomycin biosynthesis
6.	ftu00523	Polyketide sugar unit biosynthesis
7.	ftu00525	Acarbose and validamycin biosynthesis
8.	ftu00540	Lipopolysaccharide biosynthesis
9.	ftu00541	O-Antigen nucleotide sugar biosynthesis
10.	ftu00550	Peptidoglycan biosynthesis
11.	ftu00633	Nitrotoluene degradation
12.	ftu00660	C5-Branched dibasic acid metabolism
13.	ftu00680	Methane metabolism
14.	ftu00907	Pinene, camphor and geraniol degradation
15.	ftu00999	Biosynthesis of various plant secondary metabolites
16.	ftu01110	Biosynthesis of secondary metabolites
17.	ftu01120	Microbial metabolism in diverse environments
18.	ftu01501	beta-Lactam resistance
19.	ftu01502	Vancomycin resistance
20.	ftu01503	Cationic antimicrobial peptide (CAMP) resistance
21.	ftu02020	Two-component system
22.	ftu02024	Quorum sensing
23.	ftu03070	Bacterial secretion system

### Prediction of intracellular distribution

The biological activities of proteins are largely depends on their subcellular localization. It helps in comprehending its activity and disease mechanism, and in the development of new therapeutic and vaccine targets. Those proteins found in the cytoplasm were prioritized as drug targets, while those with outer membrane or extracellular sublocalizations were prioritized as vaccine targets since they can induce more robust immune responses and are readily recognized by immune cells. Both routes, KEGG-dependent and KEGG-independent, predicted 12 and 22 proteins to be in the cytoplasm, respectively. Similarly, 4 and 14 proteins were identified as outer membrane and extracellular regions, respectively.

### Vaccine target prioritization

The membranous proteins of the gram-negative bacteria *F. tularensis* were prioritized for the development of vaccines. The ProtParam Expasy server was applied to analyze the features of 18 vaccine targets, including aliphatic index, GRAVY, molecular weight, instability index, and theoretical PI. The AllerTOP v.2.0 server utilised a hybrid technique to assess protein allergenicity and pick the best vaccine candidates. The VaxiJen v2.0 server evaluated antigenicity using a > 0.5 threshold. A protein with 0 topological value was selected as the optimal vaccine target for further study (Table 2). After a thorough evaluation, A protein with the accession number WP_003035501.1 was chosen as a candidate protein and further analyzed.

**Table 2:** Vaccine Target prioritization through physicochemical properties.

Accession No.	Antigen	Allergen	Topology	Theoretical pI	Mol. wt	Instability index	Aliphatic index	GRAVY value	A.A
WP_003018671.1	Yes	No	o6-28i49-71o81-103i116-138o153-175i	9.86	21780.09	Stable	134.8	0.975	204
WP_003019784.1	Yes	No	i27-49o64-86i99-121o	6.69	14072.95	Stable	125.49	1.366	122
WP_003027262.1	Yes	No	i23-45o60-82i95-117o132-154i175-197o	6.37	22991.97	Stable	102.8	0.67	200
WP_003032934.1	Yes	No	i7-26o55-77i89-108o145-163i170-189o194-216i	9.46	25551.53	Stable	100.09	0.767	222
WP_003035501.1	Yes	No	0	5.94	46725.14	Stable	93.79	−0.311	417
WP_003040739.1	Yes	No	i9-31o64-86i99-118o131-149i162-181o191-209i	8.87	24093.78	Stable	127.7	0.826	213
WP_003038231.1	Yes	No	i12-34o49-71i117-136o	9.54	34277.52	Stable	105.71	0.103	296
WP_003038212.1	Yes	No	i20-37o72-94i114-136o140-162i247-269o293-315i351-373o393-415i428-450o460-482i503-525o562-584i	9.52	67256.86	Stable	116.71	0.563	599
WP_003038120.1	Yes	No	i7-29o	4.81	14709.53	Stable	86.07	−0.02	135
WP_003037827.1	Yes	No	i21-43o53-75i106-128o143-162i169-191o211-233i254-276o306-328i349-369o374-396i409-431o436-455i	9.58	69240.22	Stable	123.78	0.588	624
WP_003037775.1	Yes	No	i5-27o	8.27	54844.39	Stable	80.33	−0.09	509
WP_003037632.1	Yes	No	i7-29o194-211i	4.57	49264.68	Unstable	82.58	−0.56	438
WP_003037219.1	Yes	No	o32-51i	9.55	26901.67	Unstable	72.6	−0.942	235
WP_003035979.1	Yes	No	i7-29o39-61i74-96o149-166i187-209o219-241i304-326o351-373i	8.81	44421.76	Stable	141.11	1.602	416
WP_003035933.1	Yes	No	i12-34o39-58i85-107o131-153i158-180o190-221i242-264o290-312i336-358o363-385i397-419o	8.93	47723.58	Stable	146.36	1.006	420
WP_003035927.1	Yes	No	i7-29o	9.19	11168	Stable	110.52	0.076	96
WP_003035597.1	No	No	i27-49o80-97i117-139o164-186i191-208o213-232i	9.61	27455.62	Stable	107.32	0.472	239
WP_003035266.1	Yes	No	i7-27o42-64i85-104o	6.5	34527.26	Stable	91.63	0.07	300

### T-cell and B-cell epitopes

The IEDB database was used to search for MHC-binding epitopes of the chosen vaccine candidates. Epitopes with high-binding affinity were given priority. Cytotoxic T lymphocytes and Helper T lymphocytes epitopes were retrieved with the help of the NetMHCpan EL 4.0 and NetMHCIIpan EL 4.0 web server, respectively. For this purpose, the entire HLA reference allele collection from the IEDB was utilized.

The ten lowest rank Cytotoxic T lymphocytes and Helper T epitopes from each protein were also checked for antigenicity, toxicity, and allergenicity. Moreover, filtering these epitopes with various servers verified only one MHC-I binding epitope with antigenic, non-toxic, and non-allergic features (Table 3). To predict peptide interactions with MHC-II alleles, an IEDB-recommended approach with a percentile rank of less than 0.4 was utilized. The prediction of MHC class-II alleles verified two final epitopes based on antigenicity, allergenicity, and toxicity analysis (Table 3).

**Table 3:** Analysis of MHC1, MHC2, and linear B-cell epitopes.

Epitopes	Start	End	Length	Peptide	Antigenicity	Allergenicity	Toxicity
**MHC1**	83	91	9	EVLDKLFAY	Non	Non	Non
	114	123	10	LTDQKILTDY	Non	Allergen	Non
	365	373	9	ILYEPETRV	Non	Allergen	Non
	272	280	9	KVYRLGSAK	Non	Non	Non
	**223**	**231**	**9**	**RIYDSREKR**	**Antigenic**	**Non**	**Non**
	387	395	9	FIYRESQHY	Non	Non	Non
	343	352	10	SSYNSILLNK	Non	Allergen	Non
	43	52	10	AELLQSNQSF	Non	Allergen	Non
	197	205	9	HVRPAEYTK	Non	Allergen	Non
	376	385	10	FPVPPKYIGY	Non	Non	Non
**MHC2**	128	142	15	DTGIEIFANQKIHKI	Non	Allergen	Non
	304	318	15	HAKSYIQNNVVLVGD	Non	Allergen	Non
	**39**	**53**	**15**	**KEINYKSLSADRVET**	**Antigenic**	**Non**	**Non**
	214	228	15	HTAIVATIKLEKDHQ	Non	Non	Non
	320	334	15	AHTIHPLAGQGVNIG	Antigenic	Allergen	Non
	196	210	15	NSFIRDYFNFETKVK	Non	Non	Non
	398	412	15	RAGFEFVDKSRIVKS	Non	Non	Non
	68	82	15	RLGVWHSIKNKRISP	Non	Non	Non
	**169**	**183**	**15**	**SGEILNQVQDDKGLS**	**Antigenic**	**Non**	**Non**
	238	252	15	KGVLAFLPLENSNKA	Non	Non	Non
**B-Cell**	**22**	**40**	**19**	**TLDDKDKDSGPLTFPTLEP**	**Antigenic**	**Non-allergen**	**Non**
	42	64	23	TAELLQSNQSFICVKEQTGPDLI	Non	Allergen	Non
	**71**	**83**	**13**	**DADSYTLNTQAKE**	**Antigenic**	**Non**	**Non**
	108	127	20	KVESKILTDQKILTDYNIRL	Antigenic	Allergen	Non
	155	172	18	LGYQDPIAPNDSTSSRAI	Antigenic	Allergen	Non
	**295**	**327**	**33**	**NLSREASVGNYVIPNDIVSQQLPEQTFKMKSKI**	**Antigenic**	**Non**	**Non**
	335	347	13	VMNTNTFSSSYNS	Antigenic	Allergen	Non
	349	358	10	LLNKGAADGL	Non	Allergen	Non
	**369**	**381**	**13**	**PETRVDGFPVPPK**	**Antigenic**	**Non**	**Non**

The ten lowest rank MHC-I and MHC-II epitopes from each protein were also checked for toxicity, antigenicity, and allergenicity. Prioritizing seven nonoverlapping epitopes from selected antigenic proteins might lead to effective vaccine constructs against *F. tularensis*. Bepipred from the IEDB server successfully identified 13 linear B-cell epitopes obtained from the target protein sequence. Epitopes having a length higher than 10 and shorter than 40 amino acids were investigated for antigenicity, toxicity, and allergenicity, resulting in the selection of four linear B-cell epitopes (Table 3).

### Inspection of chimeric vaccine

**Fig 2:**
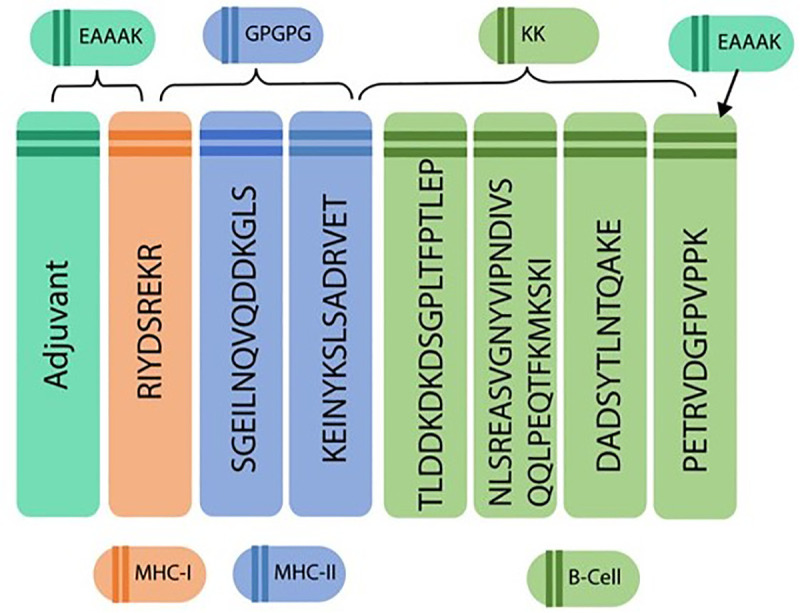
The schematical figure demonstrating the arrangement of Adjuvant, chosen CTL, and HTL epitopes in the vaccine design. The figure depicts a topographical perspective of the built final multi-epitope subunit vaccine from the selected vaccine targets.

The final epitopes were linked utilizing EAAAK, GPGPG, AAY, and KK linkers (Fig 2). The vaccine was enhanced with adjuvants that bind to the targeted epitopes, increasing its immunogenicity. Different adjuvants, i.e., beta-defensin adjuvant, flagellin adjuvant, HBHA protein, HBHA conserved sequences, and granulocyte-macrophage colony adjuvant, were added to enhance the vaccine potential. Five vaccine constructs (V1–V5) were created using various epitope and adjuvant combinations. The designed vaccines were inspected for their antigenicity, solubility, allergenicity, transmembrane helices, and physicochemical characteristics. Among them, the beta-defensin adjuvant V1 and flagellin adjuvant vaccine V2 constructs were evaluated as antigenic, non-allergenic, good solubility, with zero topology, and highly stable properties (Table 4).

**Table 4:** Physicochemical analysis of vaccines.

Vaccines	Adjuvant	Antigenicity	Allergen	Solpro	AntigenPro	Topology	A.A	Mol. wt	Aliphatic index	Gravy value	Stability
**V1**	**Beta-defensin adjuvant**	**Yes**	**Non**	**0.936448**	**0.876431**	**0**	**190**	**21029.04**	**62.63**	**−0.994**	**Stable**
**V2**	**Flagellin ADJUVENT**	**Yes**	**Non**	**0.624442**	**0.929444**	**0**	**373**	**40286.82**	**83.24**	**−0.658**	**Stable**
V3	Granulocyte-macrophage colony	Yes	Non	0.600173	0.939272	0	288	32049.45	72.5	−0.667	Unstable
V4	HBHA Adjuvant	Yes	Yes	0.905754	0.911553	0	304	33496.49	76.84	−0.777	Stable
V5	HBHA-conserved adjuvant	Yes	Non	0.905754	0.911553	0	295	32378.27	80.14	−0.752	Unstable

### 2D and 3D structures and validation

**Fig 3:**
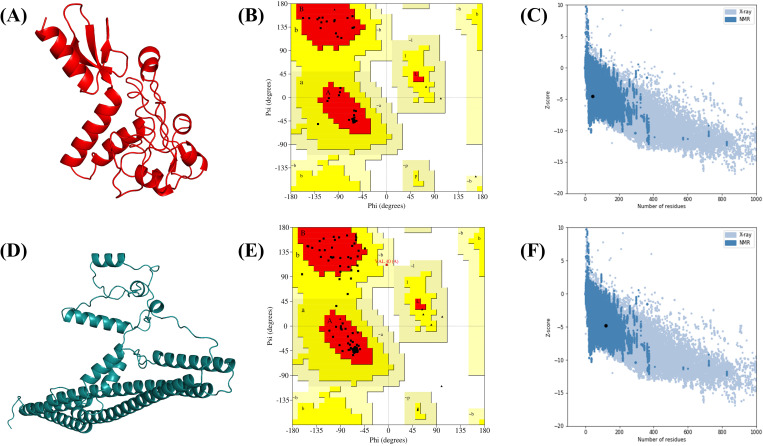
The 3D structure and validation of vaccine constructs. (A) 3D structure of vaccine V1 (B) Ramachandran plot of vaccine V1 (C) Z-score plot of V1 (D) 3D structure of vaccine V2. (E) Ramachandran plot of vaccine V2. (F) Z-score plot of V2.

The 2D structure of the constructs was determined by the SOPMA server and PDBsum. The V1 and V2 contain α-helices (23.68% to 48.87%), β-strands (4.21% to 7.89%), extended strands (20.53% to 8.65%), and random coils (51.58% to 34.59%) represented in Fig. S1 in S1 File. The 3D structures of the vaccines were built by the trRosetta. Validation of tertiary structures by the ERRAT quality factor was determined, which showed 93.75% for the V1 construct and 100% for the V2 construct. Furthermore, the Ramachandran plot identified around 97.4% residues in the favored areas of V1 and 91.6% in the V2 (Fig 3). ProSA-web was used to validate the three-dimensional (3D) model of the final vaccinations. The model with a lower Z-score was regarded to be of excellent quality. The Z-scores for the designed models of V1 and V2 were −4.50 and −4.25, respectively.

### PTM analyses

In this study, the phosphorylation locations in the vaccine constructs were determined using the web tool NetPhos3.1. The results revealed 22 phosphorylation sites (Thr: 6, Ser: 12, Tyr: 4) in the V1 and 38 phosphorylation sites (Thr: 11, Ser: 24, Tyr: 3) in the V2 vaccine construct (Fig. S2 in S1 File). Protein glycosylation is a post-translational modification that plays an important role in efficient protein folding and transportation. No glycosylation was predicted in the V1, and two positive sites in the V2 were predicted (Fig. S3 in S1 File).

### Globular regions of the vaccine

GlobPlot2 identified no globular regions in the V1 and three disordered regions: S55-G88, L103-E119, and E174-P183. Predicted disordered regions spanned some of the segments of MHC-II, MHC-I, and B-cell epitopes.

Globplot 2 analyzed two globular regions and four disordered regions: 151–184, 199–215, 270–278, and 364–369 in the V2 vaccine. The initial globular region embracedthe entire adjuvant surface and the very first B-cell epitope, whereas the second globular region roughly covered the second LBL epitope and went up to the end. Predicted disordered regions covered the CTL epitope and parts of the HTL epitope and the LBL epitope.

### Active site prediction

**Fig 4:**
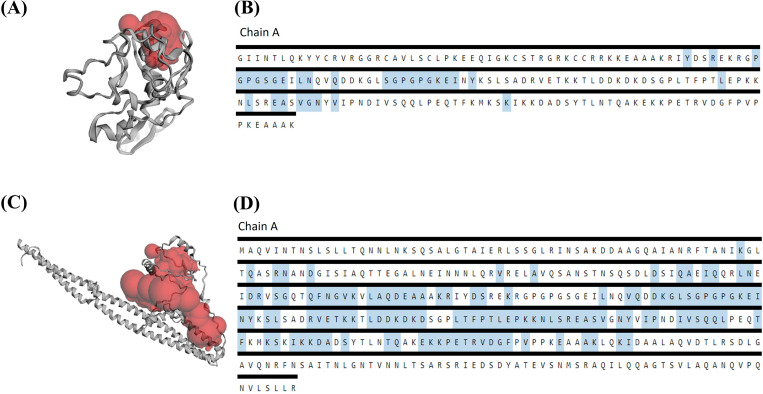
Prediction of the key residues in the vaccine constructs using the CASTp server (A) V1 (C) V2. The active amino acid residues in the vaccine constructs are marked in the diagram (B) V1 (D) V2.

The CASTp server calculated the active binding sites of the chosen vaccine constructs. It can be used as potential sites for receptor interaction. In addition, the server predicted the probable binding site with a 328.284 Å² surface area and a 261.491 Å³ molecular surface volume in V1. The maximum pocket surface area and volume were 3528.990 Å² and 9019.089 Å³, respectively, predicted in V2. This finding showed pocket volume and area were appropriate for binding (Fig 4).

### Molecular docking

**Fig 5:**
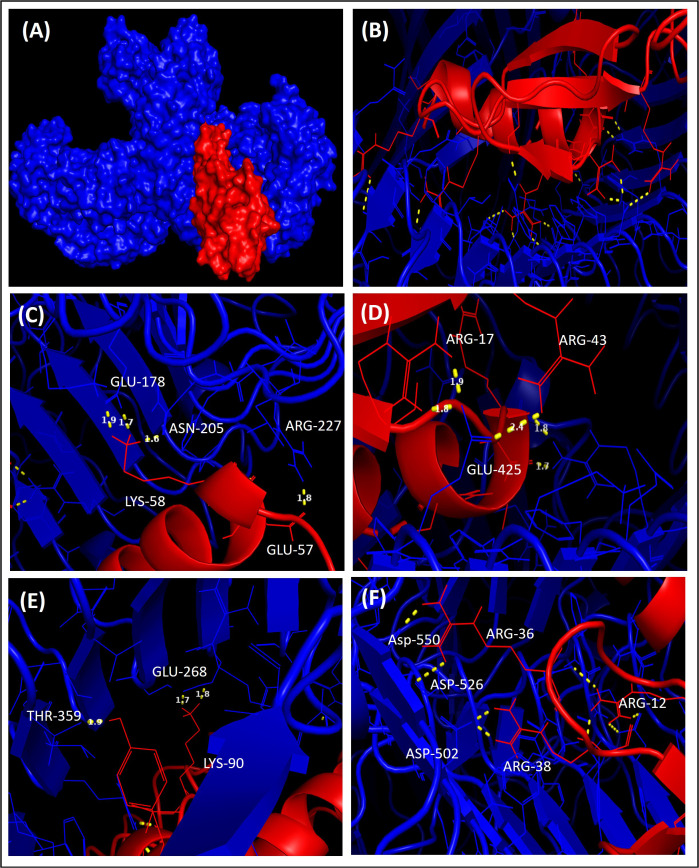
Interaction of V1 (red) with TLR4 (blue). Residues that interact with the ligand are depicted in yellow with dotted lines.

**Fig 6:**
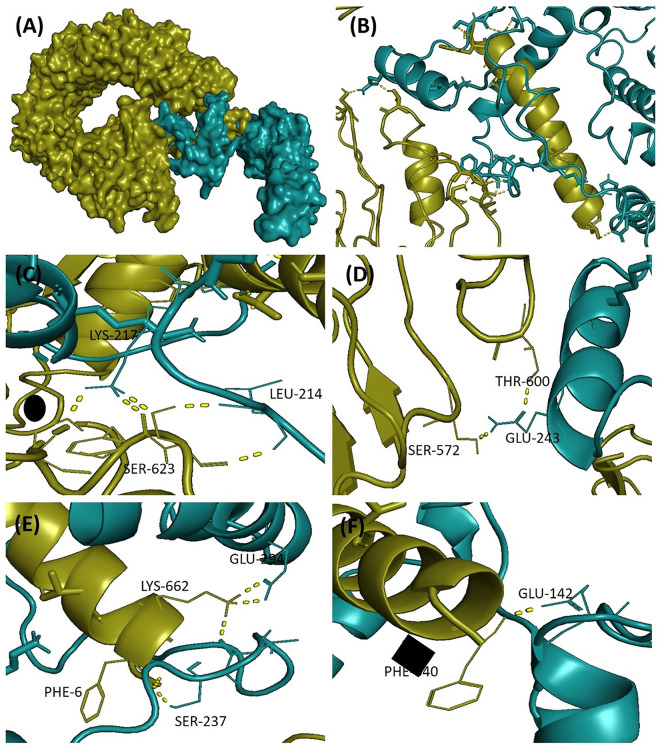
Interaction of V2 (pale green) with TLR5 (smudje). Residues that interact with the ligand are depicted in yellow with dotted lines.

V1 was docked with TLR4 receptors (PDB ID: 4G8A), and V2 was docked with TLR5 (PDB ID: 3J0A). The V1 docked with TLR4 had a docking value of −937.3 kcal/mol, whereas the V2 had docking scores of −1072.5 kcal/mol. PyMOL software was utilised for the visual representation the docked complexes and interactions between vaccine construct V1 with their respective receptor (Fig 5). The V2 had higher docking scores with immune receptors and more bonding interactions than the V1. V2 showed an interaction profile within the binding cleft of TLR5, with the maximum number of residues lying in the binding pocket (Fig 6).

### Molecular dynamic simulations

#### RMSD.

**Fig 7:**
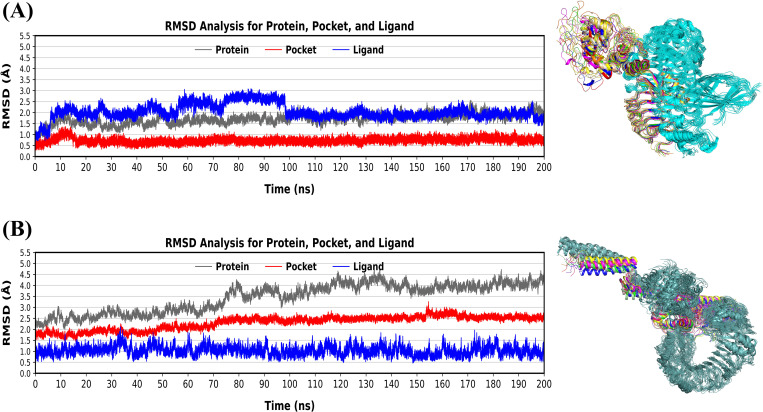
RMSD conformation values for their respective TLRs, receptor binding pocket, and ligands (A) V1 during 200 ns MD simulation (B) V2 during 200 ns MD simulation.

The Amber 20 software was used to conduct MD simulations to better recognize the stability of vaccine-TLR complexes. The RMSD was calculated to inspectthe structural stability and dynamic actions of the vaccine-receptor complexes across 200 ns simulation time. Minor oscillations in the RMSD graph indicated that the docked complexes were stable. The protein (black peaks) in the V1-TLR4 complex showed stability, with RMSD values ranging from 1 to 2.5 ± 0.5 Å, demonstrating the TLR4 structural stability during the simulation with less changes. Moreover, the binding pocket remained stable with the initial deviations, within 0.5 and 1.0 ± 0.5 Ǻ during the simulation. Furthermore, the RMSD of the ligand (blue peaks) deviated with in the moderate range of RMSD (fluctuated between 1.0 to 3.0 ± 0.5 Ǻ) in the first 100 ns. After 100 ns till 200 ns, the RMSD of the ligand remained steady ranging from 2.0 to 2.5 ± 0.5 Ǻ. The ligand showed stability V1-TLR4 system, indicating strong interactions (Fig 7A). On the other hand, in the V2-TLR5 complex (Fig 7B), the protein (black peaks) gradually increased in RMSD values from ~ 2.0 Å to 4.5 ± 0.5 Å over the simulation, indicating moderate conformational adjustments in the receptor structure while retaining overall stability. The binding pocket (red peaks) remained comparatively constant throughout the trajectory, ranging between 1.5 to 2.5 ± 1.0 Å, indicating regional flexibility without significant structural changes. The ligand (blue peaks) fluctuated minimaly and remained stable between 0.5 to 2.0 ± 0.5 Å over the simulation period of 200 ns, indicating a strong interaction inside the binding pocket. The ligand (V2) remained stable in V2-TLR5 complex, indicating a firmly bound ligand despite receptor flexibility (TLR5). Overall, both the complexes showed relative stability.

#### RMSF.

**Fig 8:**
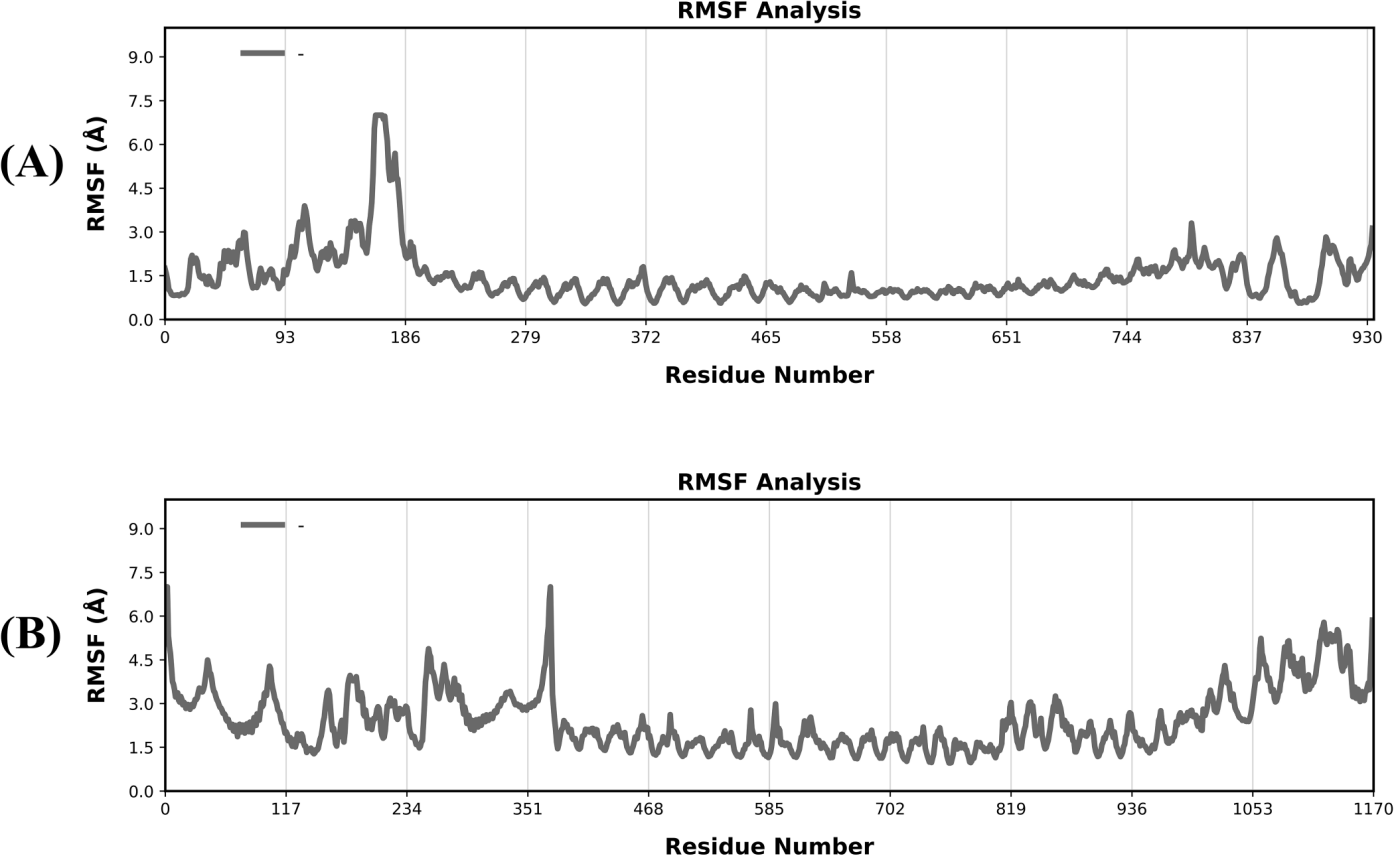
RMSF graph for each residue in the protein structure was compared for both protein-ligand complexes (A) RMSF graph for V1 acquired throughout a 200 ns MD simulation (B) RMSF graph for V2 acquired throughout a 200 ns MD simulation.

The RMSF trajectories are essential for figuring out flexibility and stability. Furthermore, to provide remarkable insights into the dynamics-function correlation resulting from the divergent evolution of protein movements, backbone C-alpha RMSFs were computed and compared. Biological processes that rely on residual flexibility or stiffness include molecular recognition, catalysis, and the binding and unbinding of biological molecules. Plot variations illustrate the dynamic and fragile character of these connections. Complex areas with less distortion and better structure are indicated by lower values or less variance. The presence of flexible regions caused significant variations in RMSF values of both complexes, i.e., V1-TLR4 (Fig 8A) and V2-TLR5 (Fig 8B). This is due to the regions that is consisted of other parts than epitopes and connect at the end terminalsPeaks on the RMSF plots represent the regions of the protein in both complexes that fluctuate the greatest during the simulation of 200 ns. The flexibility assessment of complexes on residues based was conductedfor better understanding of theirstability. Fig 8 revealed that the both the complexes had high stability and low flexibility,.

### Radius of gyration (Rg)

**Fig 9:**
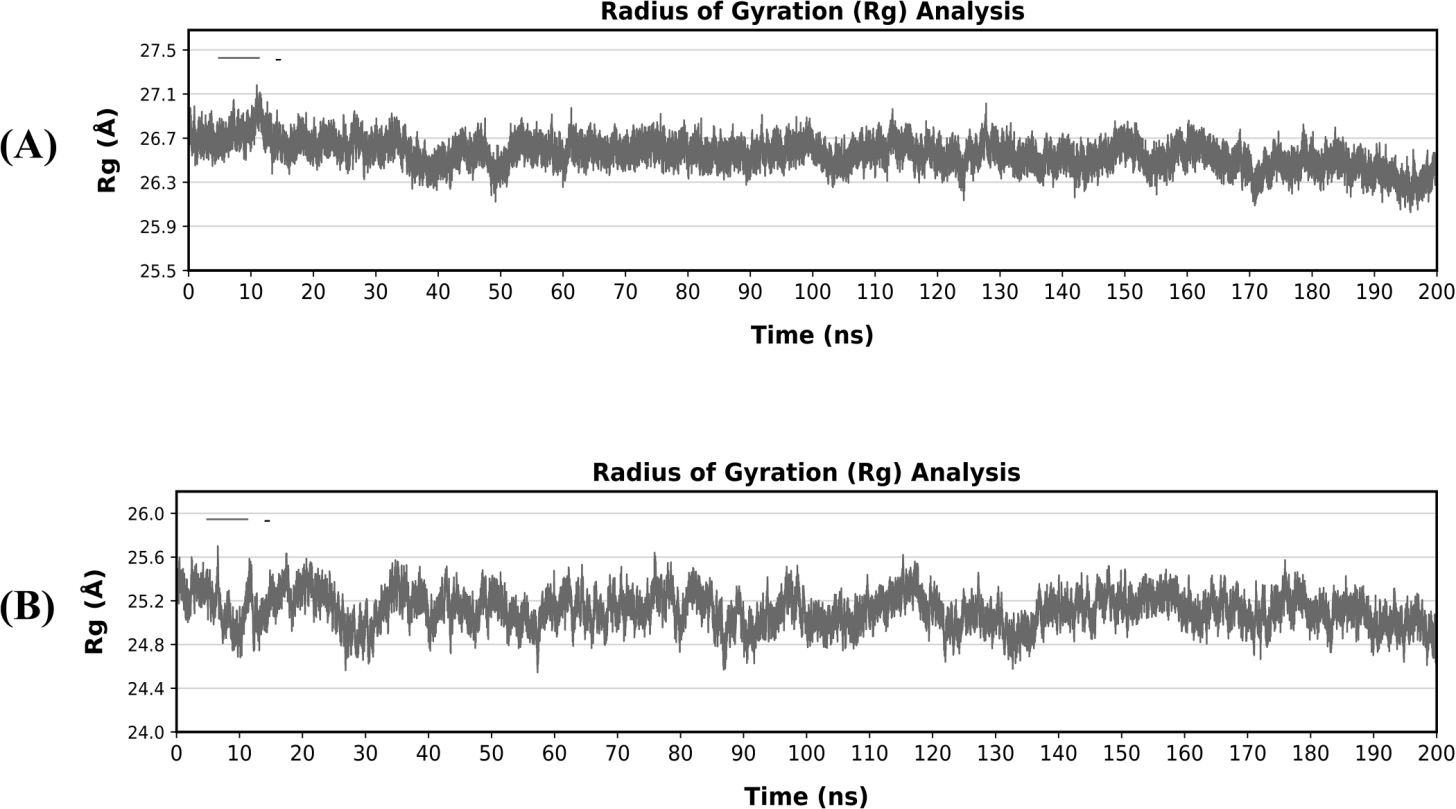
Radius of Gyration (Rg) of docked vaccine constructs with TLR4 and TLR5 (A) Rg of vaccine V1-TLR4 (B) Rg of vaccine V2-TLR5.

Additionally, the Rg for the bonded systems V1-TLR4 and V2-TLR5 was examined. Apart from the stability of protein folding, which evaluates how compact the protein is during the simulation interval, calculating the Ca atomic distribution from the center of mass is known as Rg. A steady graph depicts the protein’s stable folding, whereas a fluctuating number indicates unstable folding. The Rg peaks of V1-TLR4 (Fig 9A) and V2-TLR5 (Fig 9B) fluctuated moderately during the early equilibration phase before remaining constant in simulation period, demonstrating structural stability. Subsequent coherent Rg values in both complexes showed that protein folding was compact, supporting the results from RMSD and RMSF.

### Solvent accessible surface area (SASA) and H. bond analysis

**Fig 10:**
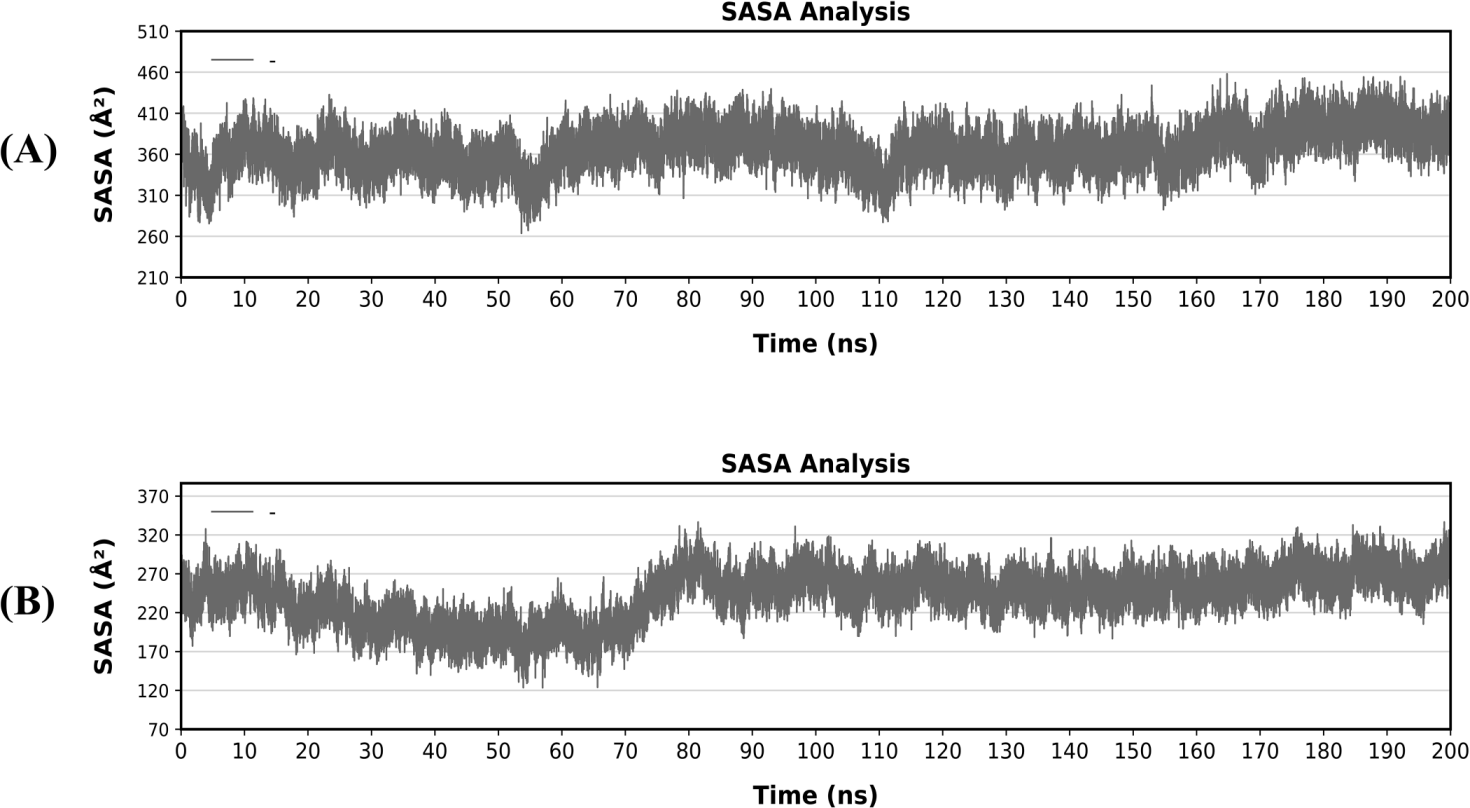
Solvent-accessible surface area (SASA) of vaccines V1 and V2 docked with TLR4 and TLR5 respectively. **(A)** SASA graph of vaccine V1-TLR4 **(B)** SASA graph of vaccine V2-TLR5.

Additionally, the solvents in the complexes to which the hydrophobic core will be exposed were estimated using the proposed SASA values for the structures. As a result, high SASA values show that a significant portion of the protein is exposed to water, whereas low values show that the hydrophobic core is protecting a significant portion of the protein. The V1-TLR4 complex’s SASA went from approximately 260 Å to 460 Å, with minor variations along the 200 ns simulation period (Fig 10A). In contrast, V2-TLR5 shows relatively constant oscillations across the course of the simulation, with a slight variation noted in the first 100 ns and then stable around 220 Å to 320 Å for 200 ns (Fig 10B). During simulation, the H-bonding interactions within vaccine-receptor complexes were examined. These interactions are critical for maintaining intramolecular stability and intermolecular sensing activities. It was also noticed that the number of hydrogen bonds increased and remained stable throughout the simulations. A pair of H-bond-forming atoms having more than 40% occupancy (Fig. S4 in S1 File).

### Binding free energy

**Fig 11:**
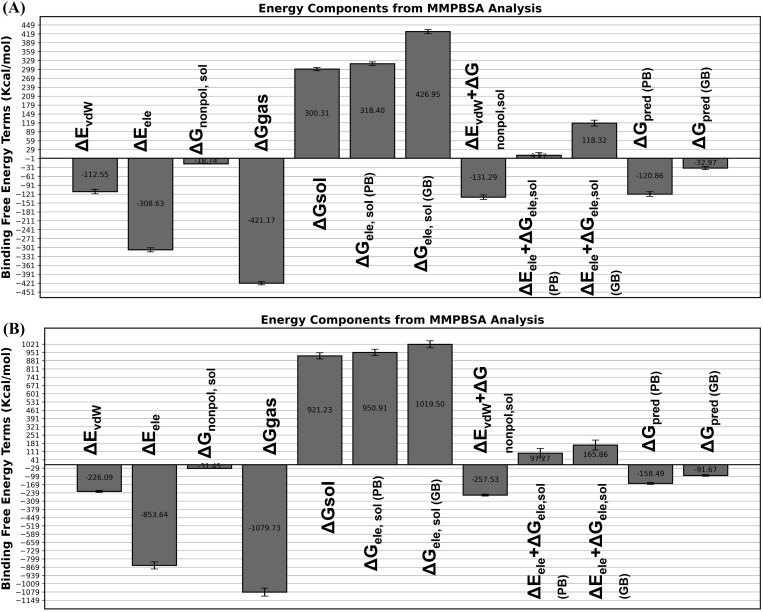
Binding free energy parameters of docked constructs during MD simulation. (A) Binding free energy plot for V1-TLR4 (B) Binding free energy plot for V2-TLR5.

The MMPBSA technique was utilized to determine binding affinities for each complex. The V1-TLR4 complex (Fig 11A) demonstrated stable binding due to favorable van der Waals interactions (ΔEvdW = −112.55 kcal/mol) and electrostatic forces (ΔEele = −308.63 kcal/mol). The non-polar solvation free energy (ΔGnonpol, sol = −18.74 kcal/mol) positively impacted complex stability. Despite polar solvation’s counteracting effects (ΔGele, sol (PB) = 318.40 kcal/mol; ΔGele, sol (GB) = 426.95 kcal/mol), the overall combination of gas-phase energy (ΔGgas = −421.17 kcal/mol) and solvation free energy (ΔGsol = 300.31 kcal/mol) maintained a robust interaction profile. The calculated binding free energies ΔGpred (PB) = −120.86 kcal/mol and ΔGpred (GB) = −32.97 kcal/mol demonstrate that the V1 vaccine candidate interacts well with TLR4, highlighting its ability to create a stable and energetically supporting complex that could boost immune responses. Moreover, the interaction between the V2-TLR5 complex (Fig 11B) was stabilized by van der Waals forces (ΔEvdW = −226.09 kcal/mol) and electrostatic energy (ΔEele = −853.64 kcal/mol), indicating substantial intermolecular attraction. The non-polar solvation free energy (ΔGnonpol, sol = −31.45 kcal/mol) enhanced binding. The polar solvation energy exhibited contrasting results (ΔGele, sol (PB) = 950.91 kcal/mol; ΔGele, sol (GB) = 1019.50 kcal/mol), but the overall balance of gas-phase and solvation energies (ΔGgas = −1079.73 kcal/mol; ΔGsol = 921.23 kcal/mol) supported a stable interaction. The projected binding free energies ΔGpred (PB) = −158.49 kcal/mol and ΔGpred (GB) = −91.67 kcal/mol validate the V2 vaccine candidate’s favorable and energetically stable complex with TLR5, indicating its significant potential to trigger successful immune activation. Furthermore, the time scale graph for MMPBSA is given in the suplemmetary figure (S5 Fig in S1 File).

### DCCM Analysis

**Fig 12:**
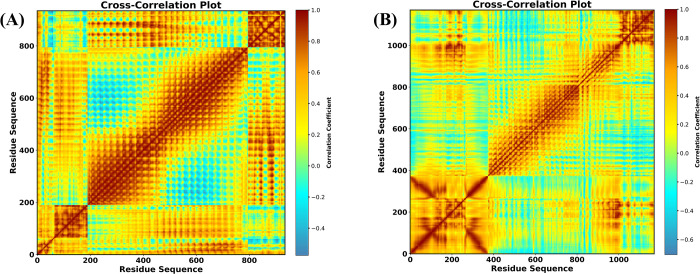
Dynamic Cross-Correlation Map (DCCM) for vaccine and TLR complex. (A) DCCM map for vaccine V1-TLR4 complex (B) DCCM map for vaccine V2-TLR5 complex.

During molecular dynamics simulations, the mobility patterns of residues inside protein complexes V1-TLR4 and V2-TLR5 are shown by the dynamic cross-correlation matrix (DCCM) (Fig 12A and 12B). The study focuses on Cα atoms’ collective motion, providing insights into their relationships and conformational dynamics. Positive correlation values (brown areas) represent residues moving in the same direction (parallel motion), while negative correlations (light blue areas) represent opposite motions (antiparallel motion).

For V1-TLR (Fig 12A), the brown areas along the diagonal, particularly between residues 200–400 and approximately 600–800, showed closely related motions, indicating structurally or functionally associated domains. Similarly, in the V2 complex (Fig 12B), prominent brown areas are observed in the range of residues 0–400 and moderately prominent brown areas in 400–800, highlighted regions of strong coordination. Moderate correlations (green and yellow areas) indicate residues with less cooperative behavior, whereas light blue zones indicate anti-correlated motions, possibly expressing antagonistic dynamics in both complexes.

Overall, these results shed light on the structural and functional mechanisms of the complexes throughout simulations, emphasizing the significance of highly correlated regions in controlling their dynamic interaction.

### PCA analysis

**Fig 13:**
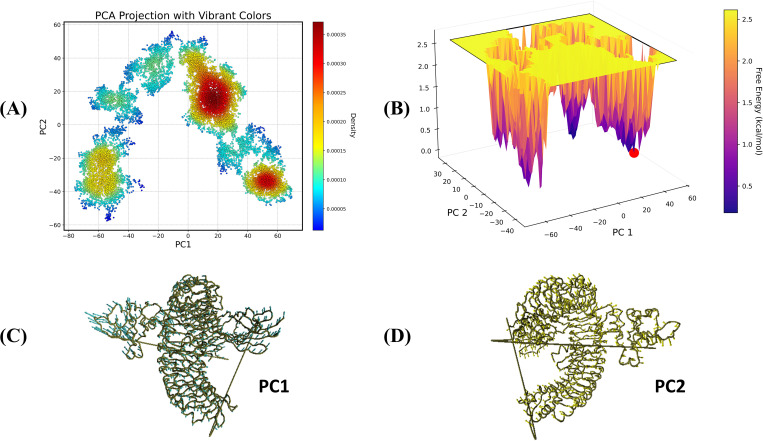
Principal component analysis and free energy landscape mapping of the V1-TLR4. (A) Blue to yellow/red color transition in the plot of PCA corresponds to the progress of the simulation with time. (B) Free energy landscape (C) PC1 and (D) PC2.

**Fig 14:**
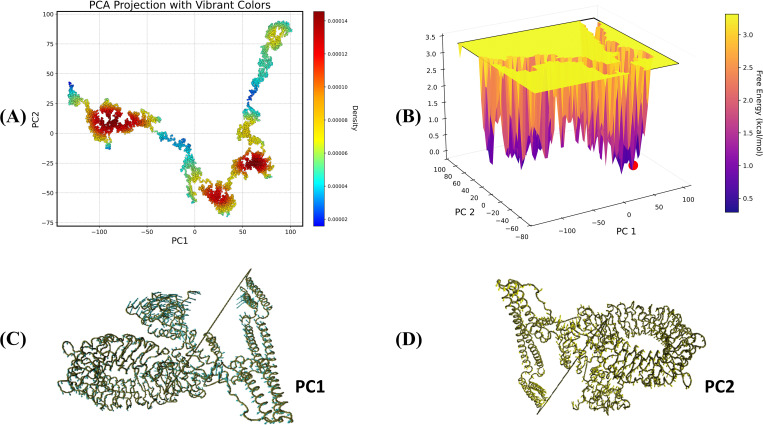
Principal component analysis and free energy landscape representation of V2-TLR5 complex. (A) In the plot, the gradient from blue to yellow/red represents the progress of the simulation with time. (B) Free energy landscape (C) PC1 and (D) PC2.

The PCA projection (Fig 13A and 14A) depicts the vaccine V1 with TLR4 and vaccine V2 with TLR5 complexes’ conformational flexibility, with PC1 and PC2 indicating the principal modes of movement. The color gradient reflects density, with red areas representing densely populated and stable structural states and blue portions representing rare and less stable states. Distinct clusters indicate the presence of multiple stable binding positions or interaction states for the complex. The free energy landscape (Fig 13B and 14B) further illustrates these states, with the z-axis denoting free energy in kcal/mol. Deep wells (purple regions) correlate to more efficient and stable conformations, which correspond to high-density regions, whereas the color red marker represents the global energy minimum, indicating the most stable V1-TLR4 (Fig 13B) and V2-TLR5 (Fig 14B) interactions. The Fig 13C & D and 14C & D represented the PC1 and PC2 of the relative complexes, responsible for the major part of the variation caused.

### NMA analysis

The iMODS service was employed to investigate the combined motions of the selected vaccine constructs by employing normal mode analysis (NMA) with the use of internal coordinates, which enables their structural stability to be evaluated. It gave detailed information regarding the dynamic behavior of structures such as V1-TLR4 complex (Fig. S6 in S1 File) and V2-TLR5 complex (Fig. S7 in S1 File), yielding extensive outputs that include eigenvalues, deformability, covariance matrices, B-factors, elastic network models, variance distributions, and residue-wise directions and magnitudes of motion.

### Discontinuous B-cell epitopes identification

The DiscoTope 3.0 and ElliPro web server were applied to investigate conformational B-cell epitopes in the ultimate vaccine constructs. DiscoTope anticipates conformational or discontinuous B-cell residues on the surface of a three-dimensional structure of a vaccine. In Fig S8A & S9A in S1 File, pink color shows that these are conformational or discontinuous B-cell epitopes. Results of Ellipro for V1: there were seven predicted conformational B-cell epitopes, with the scores varying from 0.771 to 0.567 (Table 5, Fig.S8B). For V2, there were three predicted conformational B-cell epitopes, with the scores varying from 0.78 to 0.69 (Table 6, Fig. S9B in S1 File).

**Table 5:** The B cell epitopes identified by Ellipro in the structure of V1.

Sr No.	Residues	Number of residues	Score
1	A:G1, A:I2, A:I3, A:N4, A:T5, A:L6, A:Q7, A:K8, A:Y9, A:Y10, A:C11, A:R12, A:V13, A:R14, A:G15, A:G16, A:C18, A:A19, A:V20, A:L21, A:S22, A:C23, A:Q29, A:G31, A:K32, A:C33, A:S34, A:T35, A:R36, A:G37, A:R38, A:K39	32	0.771
2	A:Y162, A:T163, A:L164, A:N165, A:T166, A:Q167, A:A168, A:K169	8	0.77
3	A:D54, A:S55, A:R56, A:E57, A:K58, A:R59, A:G60, A:P61, A:G62, A:P63, A:G64, A:S65	12	0.704
4	A:S141, A:Q142, A:Q143, A:L144, A:P145, A:E146, A:K150, A:K172, A:P173, A:E174, A:T175, A:R176, A:V177, A:D178, A:G179, A:F180, A:P181, A:P183, A:P184, A:K185, A:E186	21	0.672
5	A:K122, A:N123, A:R126	3	0.642
6	A:L103, A:K106, A:D107, A:K108, A:D109, A:S110, A:G111, A:P112, A:L113	9	0.591
7	A:S79, A:G80, A:P81, A:G82, A:P83, A:G84, A:K85, A:E86, A:I87, A:N88, A:K90, A:E119, A:P120, A:K121	14	0.56

**Table 6:** The conformational B-cell epitopes of the V2 identified by Ellipro.

Sr No.	Residues	Number of residues	Score
1	A:K197, A:D201, A:K204, A:D205, A:S206, A:G207, A:P208, A:L209, A:T210, A:F211, A:P212, A:T213, A:L214, A:E215, A:P216, A:K217, A:K218, A:N219, A:L220, A:S221, A:R222, A:E223, A:A224, A:S225, A:V226, A:G227, A:N228, A:Y229, A:V230, A:I231, A:P232, A:N233, A:D234, A:I235, A:V236, A:S237, A:Q238, A:Q239, A:L240, A:P241, A:E242, A:Q243, A:T244, A:F245, A:K246, A:M247, A:K248, A:S249, A:K250, A:I251, A:K252, A:K253, A:D254, A:A255, A:D256, A:S257, A:Y258, A:T259, A:L260, A:N261	60	0.789
2	A:M1, A:A2, A:Q3, A:V4, A:I5, A:N6, A:T7, A:N8, A:S9, A:L10, A:S11, A:L12, A:L13, A:T14, A:Q15, A:N16, A:N17, A:L18, A:N19, A:K20, A:S21, A:Q22, A:L25, A:S335, A:D336, A:Y337, A:A338, A:T339, A:V341, A:S342, A:N343, A:M344, A:S345, A:R346, A:A347, A:Q348, A:I349, A:L350, A:Q351, A:Q352, A:A353, A:G354, A:T355, A:S356, A:V357, A:L358, A:A359, A:Q360, A:A361, A:N362, A:Q363, A:V364, A:P365, A:Q366, A:N367, A:V368, A:L369, A:S370, A:L371, A:L372, A:R373	61	0.782
3	A:R91, A:V92, A:R93, A:E94, A:L95, A:A96, A:V97, A:Q98, A:S99, A:A100, A:N101, A:S102, A:T103, A:N104, A:S105, A:Q106, A:S107, A:D108, A:L109, A:D110, A:S111, A:I112, A:Q113, A:A114, A:E115, A:I116, A:Q118, A:R119, A:V273, A:D274, A:G275, A:F276, A:P277, A:V278, A:P279, A:P280, A:K281, A:E282, A:A283, A:A284, A:A285, A:K286, A:L287, A:Q288, A:K289, A:I290, A:D291, A:A292, A:L294, A:A295, A:D298	51	0.695

### Immune simulations

The chosen vaccine constructs, V1 and V2, were tested for immune response simulation through the C-ImmSim server to determine their capability to elicit human immune responses after a period of time. The modeled vaccination regimen consisted of three doses given at 1, 84, and 168 hours. With every subsequent dose, immunogenicity grew and peaked following the third injection. The vaccine triggered the activation of Tc-cells, B-cells, Th-cells, and NK-cells. In V1, IgM and IgG levels increased enormously following the last dose (Fig. S10 in S1 File). Also, V2 induced the formation of several types of antibodies such as IgG1, IgM, IgG, and IgG2 (Fig. S11 in S1 File). Moreover, the computer-simulated vaccine injections revealed very low risk of immunologically adverse reactions.

### In-silico cloning

**Fig 15:**
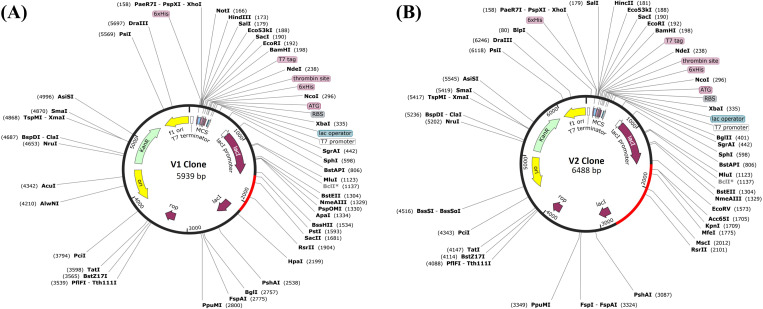
The image depicts in silico vaccine cloning. (A) V1 (B) V2 in an Escherichia coli pET28a (+) vector, with the vaccine highlighted in red.

The constructed vaccine candidates had a GC content between 0% and 50% and a Codon Adaptation Index (CAI) score of 1.0, indicating effective expression in E. coli (K12 strain). To promote cloning, codon-optimized V1 and V2 were subcloned into the pET28a(+) plasmid vector using SnapGene software. The obtained recombinant vectors harbored the vaccine DNA sequences, establishing successful cloning into pET28a(+) and allowing their heterologous expression in host cells (Fig 15).

### Population coverage analysis

**Fig 16:**
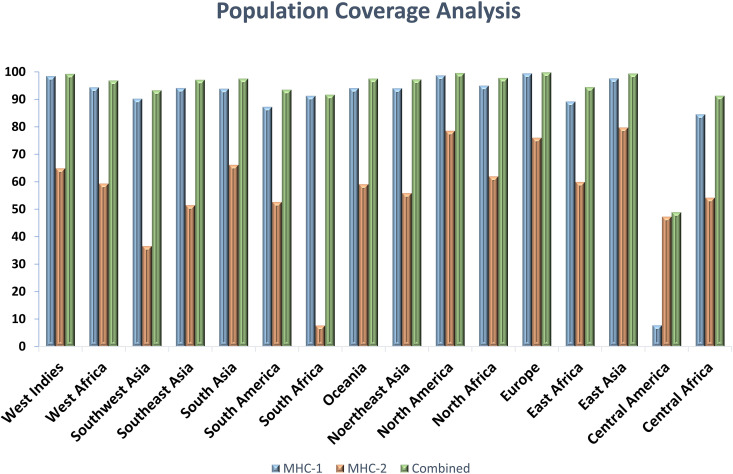
Worldwide population coverage graph of the epitopes involved in the MEV based on their respective HLA binding alleles.

HLA allele frequencies differ in populations, and it is therefore important to examine HLA compatibility with desired vaccine epitopes to guarantee effectiveness in various populations. Predicting population coverage using the IEDB population coverage tool, people from countries are expected to respond well to chosen T-cell epitopes due to HLA compatibility (Fig 16).

## Discussion

*Francisella tularensis* is an intracellular bacterium with high virulence for the zoonotic disease tularemia. It is categorized under re-emerging infections and has undergone numerous recent outbreaks in various parts of the world [1]. Tularemia is treated with a few antibiotics, including tetracyclines (e.g., doxycycline), fluoroquinolones (e.g., ciprofloxacin), and aminoglycosides (streptomycin and gentamicin) [10]. But due to the development of antibiotic-resistant F. tularensis is a challenging one [11]. In the absence of presently available licensed and safe vaccines, the need to develop vaccines against this pathogen is immediate. Targeting the core genome of the bacterium is seen as a potential strategy for identifying new therapeutic compounds. Here, bioinformatics-based in-silico methods are being used increasingly to search for promising drug and vaccine candidates against such pathogenic bacteria.

In this research, a subtractive proteomics strategy was used to screen for proteins that are antigenic, critical, human protein non-homologous, and non-toxic; hence, they are excellent candidates for vaccine development against Francisella tularensis. One of the major selection criteria in target identification was the lack of transmembrane helices. On the basis of these considerations, one protein, alone OmpA family protein (Accession No: WP_003035501.1) was chosen as the vaccine target. The VaxiJen v2.0 server was utilized to evaluate the protein’s antigenicity, while Allertop was used to examine its allergenicity. To invoke humoral and cellular immune responses, it is important that B and T cell epitopes collaborate to produce a robust and long-term immune response. Thus, thorough B and T-cell epitope prediction and analysis were done. Population coverage analysis also revealed that the chosen epitopes were likely to trigger an immune response in 94.87% of the world’s population [54].

Three linkers were included to connect the chosen epitopes in the vaccine construct. The AAY (Ala-Ala-Tyr) linker, which is known as a mammalian proteasomal cleavage site, supports intracellular processing and reduces junctional immunogenicity, finally delivering more immunogenic potential towards multi-epitope vaccines [55]. Because the KK (di-lysine) linker prevents antibodies from forming against linear epitope sequences, it lowers junctional immunogenicity [56]. In addition, KK linkers have been reported to enhance the general immunogenic status of the vaccine construct [57]. The GPGPG linker, which is made up of glycine and proline residues, is also very effective in stimulating helper T lymphocyte (HTL) responses, which are pivotal to the efficacy of multi-epitope vaccines. It also facilitates the preservation of individual immunogenicity of the joined epitopes by preventing junctional immunogenicity [58]. To further enhance immunogenicity, adjuvants were linked to both the N- and C-termini of the construct by using the α-helical EAAAK linker. This linker acts as a stiff spacer that ensures appropriate structural conformation, increases the biological activity and expression of the fused protein [59]. Adjuvants are most important in vaccines since they enhance immune responses and augment the overall effectiveness of the formulation. Five different adjuvants were used in this study: β-defensin, flagellin, granulocyte-macrophage colony-stimulating factor (GM-CSF), heparin-binding hemagglutinin adhesin (HBHA), and HBHA-conserved peptides to produce vaccine constructs with different immunological properties.

β-defensin was chosen due to its reported capacity to induce both humoral and cellular immunity. It serves as both an antimicrobial peptide and an immunomodulator, recruiting dendritic cells and T lymphocytes via CCR6 signaling, contributing to its superior efficacy. Its adjuvant effect has already been proven in experimental vaccines [60,61]. Flagellin was used because it has the ability to increase antigen-specific responses and facilitate effective antigen presentation through dendritic cells [62]. Flagellin is a well-studied ligand for Toll-like receptor 5 (TLR5) that activates innate immunity and elicits strong adaptive immunological responses. It has been widely employed as a molecular adjuvant and has proved to improve antigen-specific antibody titers and T-cell responses in subunit and peptide-based vaccinations [63,64]. Granulocyte-macrophage colony-stimulating factor (GM-CSF) was added because it supports the dendritic cell maturation, increases antigen presentation, and amplifies humoral and cellular immunity [65]. GM-CSF facilitates CD4 + T cell differentiation into helper subsets, thus enhancing cytotoxic T lymphocyte (CTL) responses [66]. Heparin-binding hemagglutinin (HBHA) has been shown to highly upregulate inflammatory cytokines and adhesion molecules. HBHA was added due to its capacity for facilitating adhesion and T-cell mediated immunity, and hence serving as a possible vaccine adjuvant [67]. HBHA-conserved peptide adjuvant was added to assess the ability of conserved immunodominant regions to improve immunogenicity [68]. Two of the constructed vaccines using β-defensin and flagellin as adjuvants showed desirable features, such as high antigenicity, non-allergenicity, solubility, and a stable topology score of zero. The final vaccines, V1 and V2, both had grand average hydropathicity (GRAVY) values of −0.994 and −0.658, confirming their hydrophilic nature. This allows for better interaction with the polar ecosystem. Based on bioinformatic techniques, the molecular weight of the candidate vaccines of V1 and V2 has been determined to be 21 kDa and 40.2 kDa, which, in comparison to the optimum circumstances (less than 110 kDa), is regarded as an appropriate and efficient vaccine [69]. Additionally, MEVC exhibits a considerably higher aliphatic index, a measure of protein stability, suggesting increased resistance to proteolytic degradation. In addition, the instability index, a parameter indicating protein stability, shows a large difference between the two vaccine candidates. The fact that this value of the instability index is lower in MEVC implies a higher chance of structural integrity at storage and transport, an essential consideration in vaccine production.

MEVCs possess a good-quality 3D structure derived from structural modeling and optimization. The good-quality factor from ERAAT of 93.75% for the V1 construct and 100% for V2 construct, over 91.6% residue distribution to the allowed area of Ramachandran plot for V1 and 97.4% for V2 complex, and good Verify3D results, all depicting a good-folded and trustworthy protein structure, validate its quality. ERRAT is a unique approach that may discover inaccurate portions of protein structures based on errors resulting in random distributions of atoms, which can be separated from a correct distribution [70]. Ramachandran plots, which characterize protein structures by graphing the dihedral angles of the backbone on a two-dimensional plane, indicate the structural stability of proteins [71]. MEVC’s ability to be an effective vaccine candidate needs a stable and well-folded structure, which supports optimal presentation of epitopes and effective immune recognition. In addition, a reliable tertiary structure is important for optimizing and rational vaccine design [13].

Toll-like receptors (TLRs) are the key molecules for the detection of viral elements and activation of innate immunity. Because of their pivotal role, TLRs are attractive targets for vaccine development and disease management strategies. Molecular docking and molecular dynamics (MD) simulations at 200 ns were utilized in this study to examine the final multi-epitope subunit vaccine’s binding affinity and mechanisms of interaction with TLR4 and TLR5. TLR4 is expressed on numerous immune cell types (macrophages, monocytes, DCs, and granulocytes); hence, associated immunization will give adequate immune responses [71]. While the flagellin protein serves as a TLR5 agonist, TLR agonists play an important role in stimulating innate and adaptive immunity [72]. Stability analysis through MD simulations verified that the vaccine-receptor complexes were stable through the simulation period [73].

Since prokaryotes and eukaryotes have different codon usage, codon optimization is necessary to produce eukaryotic proteins effectively in *E. coli*. The reason behind this is that prokaryotic systems like *E. coli* do not translate synonymous codons of a codon family with the same efficiency [74]. The *E. coli* K12 strain was used to optimize codons for high-level protein expression. With a GC content of 50% and a Codon Adaptation Index (CAI) of 1.0, the improved sequence demonstrated remarkable translational efficiency. These findings imply that the vaccine design was effectively expressed within the *E. coli* system, demonstrating the effective implementation of the expression process [75].

Further, immune simulation of the designed vaccine also indicated strong humoral and cellular immune responses. Overall, the bioinformatics and immunoinformatics analyses are highly supportive of the likely effectiveness of this vaccine candidate against *F. tularensis*. Nonetheless, it should be mentioned that while computational approaches are highly potent in providing initial data, the predictability and accuracy of these tools are constrained by algorithms. Experimental confirmation through in vitro and in vivo experiments is thus required to validate the effectiveness of the vaccine.

## Conclusion

Herein computational methodologies were employed to identify new therapeutic targets for *F. tularensis*, which currently lacks an effective vaccine to prevent infection. A computational screening of 72 entire genomes using updated biological datasets revealed 18 novel membrane vaccine targets that were not previously described. T-cell and B-cell epitope predictions were then employed to create a vaccine construct from the prioritized protein. Five vaccine designs (V1-V5) were created using various epitope and adjuvant combinations. V1 and V2 are identified as the most effective architectures for activating the human immune response. This design may elicit a human immunological response, as the stability of MEV constructs was also checked by docking and simulation analysis. The vaccine was cloned in silico on *the E. coli* strain K12 bacterium to confirm its cloning potential. Immunoglobulins bind more strongly to this design, as validated by immunological simulation research. To summarize, it is believed that the suggested vaccine designs are ready for further testing to establish their biological efficacy against *F. tularensis* infections.

## Supporting information


S1 File.
**S1 Fig.** 2D structure of vaccine constituting alpha-helix, beta-turn, and coils. **(A)** 2D structure of vaccine V1 **(B)** 2D structure of vaccine V2. **S2 Fig.** Post-translational modification of vaccine construct. **(A)** Phosphorylation sites of vaccine V1 **(B)** Phosphorylation sites of vaccine V2. **S3 Fig.** Post-translational modification of vaccine construct. **(A)** Glycosylation sites of vaccine V1 **(B)** Glycosylation sites of vaccine V2. **S4 Fig.** Hydrogen bond analysis of the V1-TLR4 and V2-TLR5 complexes. **S5 Fig.** The time scale graph for MMPBSA during the MD simulation. **(A)** V1-TLR4 **(B)** V2-TLR5. **S6 Fig.** Normal mode analysis (NMA) evaluation of vaccine construct V1**(A)** Covariance (B) Variance **(C)** E-value **(D)** Elastic Network **(E)** Deformability **(F)** B-Factor. **S7 Fig.** Normal mode analysis (NMA) evaluation of vaccine construct V2**(A)** Covariance **(B)** Variance **(C)** E-value **(D)** Elastic Network **(E)** Deformability **(F)** B-Factor. **S8 Fig.** Confirmational B-cell epitopes of vaccine V1. **(A)** Confirmational B-cell epitopes of vaccine V1 through Discotop 3.0 **(B)** Confirmational B-cell epitopes of vaccine V1 through Ellipro. **S9 Fig.** Confirmational B-cell epitopes of vaccine V2. **(A)** Confirmational B-cell epitopes of vaccine V2 through Discotop 3.0 **(B)** Confirmational B-cell epitopes of vaccine V2 through Ellipro. **S10. Fig.** Immune simulation of vaccine V1 by C-Immsim Server. **(A)** Interleukins **(B)** B-cell population per state **(C)** Antigen Count **(D)** TH cell population per state **(E)** TC cell population per state **(F) NK** cell population per state. **S11 Fig.** Immune simulation of vaccine V2 by C-Immsim Server. **(A)** Interleukins **(B)** B-cell population per state **(C)** Antigen Count **(D)** TH cell population per state **(E)** TC cell population per state **(F)** NK cell population per state.
(RAR)
